# Supplementary material for: Report: The effects of topical pleurotus tuberregium (PT) aqueous extract on intraocular pressure in monkeys
Source: PLoS One. 2021 Aug 24;16(8):e0256422. doi: 10.1371/journal.pone.0256422 (PMC8384159; doi:10.1371/journal.pone.0256422)
Supplement: Attachment — Submitted filename: George Vousden letter to Plos one-PK-10-23-20GA.docx [file pone.0256422.s001.docx]

Top of Form

[<https://www.google.com/imgres?imgurl=https%3A%2F%2Fwww.uniben.edu%2Fassets%2Fimg%2Flogo2.png&imgrefurl=https%3A%2F%2Fwww.uniben.edu%2F&tbnid=FOicd2rOZFmHCM&vet=12ahUKEwi58ejs8LPsAhVCZRoKHR_xAQIQMygAegUIARCfAQ..i&docid=5e0uOZB9H6VFDM&w=650&h=480&q=uniben%20logo&client=safari&ved=2ahUKEwi58ejs8LPsAhVCZRoKHR_xAQIQMygAegUIARCfAQ> Department of Optometry, Faculty of Life Sciences](https://www.google.com/imgres?imgurl=https%3A%2F%2Fwww.uniben.edu%2Fassets%2Fimg%2Flogo2.png&imgrefurl=https%3A%2F%2Fwww.uniben.edu%2F&tbnid=FOicd2rOZFmHCM&vet=12ahUKEwi58ejs8LPsAhVCZRoKHR_xAQIQMygAegUIARCfAQ..i&docid=5e0uOZB9H6VFDM&w=650&h=480&q=uniben%20logo&client=safari&ved=2ahUKEwi58ejs8LPsAhVCZRoKHR_xAQIQMygAegUIARCfAQ)

October 15, 2020.

George Vousden, PhD
Staff Editor
PLOS ONE

[em@editorialmanager.com](mailto:em@editorialmanager.com)

Dear Dr. Vousden,

Thank you for considering my submitted manuscript to PLOS ONE for review. We are compliant with all the issues raised, as indicated below:

Issues raised:
‘In your submission, you have not provided us with sufficient details regarding the animal research study design, rationale, methodology, or results to allow for internal evaluation of whether your study meets these standards and can be sent for peer review.

It is not clear whether your institutional animal ethics committee specifically reviewed and approved the procedures in the study. Furthermore, details regarding basic animal husbandry, housing, and efforts to alleviate harm and suffering have been outlined in sufficient detail. We are also concerned that details of chemical characterization or authentication of the product used, and quantification of the dosage have not been provided’.

The answers are now in the revised manuscript and below.

Answers:

The rationale for the study was to find out whether the extract can lower intra ocular pressure in normal non-human primates, as it did in our earlier studies in dexamethasone-induced ocular hypertensive cats. This is now stated on lines 70-72 in the manuscript (MS).

A copy of the excel spreadsheet used in the detailed analysis of the result is attached.

The study was carried out in Professor Paul Kaufman’s laboratory, which is housed within the Wisconsin National Primate Research Center (WNPRC), one of the seven NIH-supported national primate research centers in the US, situated on the University of Wisconsin-Madison campus. Dr. Kaufman’s is a world-renowned laboratory for primate research in glaucoma, situated within the world-class facilities of the WNPRC that adhere to the highest standards of animal husbandry, and veterinary care and oversight. Indeed, they are the global paradigm. The animals are housed literally down the hall from the laboratory. All personnel must pass numerous annual training certifications. A University of Wisconsin institutional animal care and use committee (IACUC) reviewed and approved the study and all its procedures. All necessary and approved efforts were deployed to prevent, minimize and alleviate any harm or suffering for the animals. A UW-Madison IACUC protocol approval is attached (see pages 10, 47 etc.). This is now summarized in the revised MS, - lines 97-102.

Authentication of the product used (Pleurotus tuber-regium (PT)) was done by Professor Omon Isikhuemen of the North Carolina A & T State University, Greensboro, NC, USA. The quantification of the dosage was stated as: ‘Powdered PT (27.217 g) from Nigeria was used to prepare the PT suspension in Dr. Paul Kaufman’s lab. The powder was combined with 320 ml distilled water and the mixture was stirred for 3 days, at room temperature, using a Nuovo stirrer. The mixture was then filtered using a Nalgene MF75 series polystyrene Lab Filter unit, with a pore size of 0.45 µm. The filtered extract (150 ml) was heated and stirred until the liquid evaporated, yielding 3.03 g of a brown solid mass. The final yield was diluted using distilled water to either 10 mg/ml or 50 mg/ml and had a pH of 4.3. The diluted extract was sterilized using an autoclave’. The monkeys were treated twice daily for 4 days with 2 x 20 µl drops of 50 mg/ml PT (at 1- minute interval between the drops). This is in the revised MS- lines-84-90, 113-115, and 121-123.

Thanks again for helping us with the proper procedures.

Yours sincerely,
G.A. Akinlabi
