# Supplementary material for: Report: The effects of topical pleurotus tuberregium (PT) aqueous extract on intraocular pressure in monkeys
Source: PLoS One. 2021 Aug 24;16(8):e0256422. doi: 10.1371/journal.pone.0256422 (PMC8384159; doi:10.1371/journal.pone.0256422)
Supplement: Attachment — Submitted filename: RESPONSE TO REVIEWERS 1-PKGAPKGA4-3 - PK-2GA12-3 - PKGA14-3.docx [file pone.0256422.s002.docx]

RESPONSE TO REVIEWERS

Thank you for favorably considering our manuscript and for the corrections pointed out therein.

Below are the answers to the questions raised by the reviewers.

Issues raised by academic editor

The study on topical use of aqueous extract of Pleurotus tuberregium mushroom on intra-ocular pressure in monkeys is a interesting study. However, there are some concerns in the study which is elaborated in the comments of the reviewers. Moreover, the authors have studied only the effect of Aqueous extracts, which contains only glucans and proteins but alkaloids, saponins, flavonoids, tannins, anthraquinnone, etc can not be extracted in water extracts. Thus the study do not represent the complete effect of PT.

Response

We will study the effect of ethanolic extract on IOP. It has been reported that phytochemical analysis of ethanolic and aqueous extract of P. tuber-regium reveals that traces of polyphenols and saponins were more in ethanolic than aqueous extract, although alkaloids, glycosides, saponins, flavonoids, tannins and polyphenol, were present in both aqueous and ethanolic extract^41^ (line 227 - 230).

Reviewer #1: 

1. In the introduction you mention "...Scientific evidence indicates that PT is useful in treating ... ". The references provided after that are only for the in vitro and in vivo animal works. Please provide references to placebo-controlled clinical trials to support this statement. Otherwise it is highly misleading.

Response

The sentence has been changed to ‘Scientific evidence indicates that PT is useful in treating high blood pressure, diabetic hypertriglyceredemia, tumours, as well as fungal and bacterial infections in animals and humans^4-15^, but to our knowledge no randomized masked placebo-controlled trials have been reported.’ (line 55-58)

2. The scientific premise for the work is not clear. There is no suggested mechanism of action for the PT extract in lowering IOP.

Response

Mechanistic studies will be carried out later. PT was reported to contract muscarinic receptor-containing bovine iris muscle in an organ bath preparation^19^. However, it must be remembered that the iris has nothing to do with aqueous outflow. In live monkeys, the iris can be completely removed, and IOP, outflow facility, and the facility response to pilocarpine are unchanged^23,24,25^. If there is muscarinic receptor-mediated iris muscle contraction, there could also be muscarinic receptor-mediated contraction of the ciliary muscle in monkeys, and that might increase facility. Aside from that we are agnostic as to the mechanism. Assuming that PT lowers IOP in live NHP, we would identify the individual physiological parameter affected (aqueous humor formation, conventional outflow, uveoscleral outflow, Schlemm’s canal and episcleral venous pressure, etc. These parameters can each be measured ^26-30^. (Line 180-190).

3. What standardization and quality control procedures were used to normalize PT preparations and guarantee reproducibility of the studies?

Response

Mushroom crops were produced in shade houses, harvested by hand and sundried. All water used in cleaning and processing were purified with an ultra-filtration system.This super fine membrane technology filters particulate down to 0.025 microns. (Line 86-88).

4. For the IOP measurements - what time of day were they performed? Could you also describe the housing of animals and their expected circadian cycle?

Response
IOP measurements started at 9 am after treatment and every hour after that, for 3 hours (line 114).

The monkeys were kept in standard AALAC- and USDA-approved WNPRC housing and husbandry conditions, under a 12/12hour light/dark cycle. (Line 115-117).

5. In the discussion you make the conclusion that that the "extract has no effect on the IOP in normal eyes" and also make the statement that "there is a possibility that the study groups were small to detect any effect." These statements are contradicting - please correct, with the statistical power if needed.

Response

The wording has been changed to:

The extract had no effect on the IOP in our small group of normal eyes, but there is a possibility that the study group was too small to detect a small effect. Sample size calculation for paired organs conducted based on the mean and standard error of the current data indicated that at n equals 5 to 8, we would have sufficient power to detect a physiologic response greater than or equal to 25% of baseline for a two-sided test and 5% significance^22,23^. The calculation was for outflow facility; theoretically, IOP might be different, although IOP is linked to outflow facility. While our data set is small, this preliminary experiment tells us that we are not likely missing a big effect that occurs across the board in all animals. Whether a higher dose or longer duration would have mattered we cannot say, but this is not like pilocarpine, epinephrine, rho kinase inhibitors, timolol, nitric oxide donators, PGF_2⍺_ analogues, etc., where there is a big effect after short-term treatment in most human or monkey subjects^31-40^. (Line 180-190).

6. What is the expected mechanism of action for the PT extract?

Response

The contractile effect of PT on muscarinic receptor-containing iris muscle may explain its IOP reducing property^19^. If there is muscarinic receptor-mediated iris muscle contraction, there could also be muscarinic receptor-mediated contraction of the ciliary muscle in monkeys, and that might increase facility. This is only one *possible* mechanism; any of the other parameters affecting aqueous humor formation or outflow could be at play. One has to start somewhere……](Line 179 – 186).

7. There are several factors that contribute to IOP regulation - could you elaborate on the actual effect of PT extract on the aqueous humour production/outflow, TM state, etc. Also, do you expect it to have different effect in dexamethasone-induced glaucoma and healthy eyes?

Response

Earlier work shows that PT extract reduces IOP in dexamethasone-induced glaucoma in a feline model^20,21^. Mechanistic studies would be needed to uncover an effect of PT on aqueous humor production/outflow (trabecular meshwork/ Schlemm’s canal. EVP, uveoscleral outflow, etc.), and then to analyze morphology/ultrastructure, etc. in primates. This is far, far beyond the scope of the current exploratory study. (Line 222 – 226)

Reviewer #2: Manuscript is good
Few typographical or grammatical errors need to be checked
Such as in abstract Line no 27. Correct the spelling of Pleurotus tuberregium
Similarly line no. 54 lignocellulosic in place of lognocellulosic

Correct these small errors

Response

The errors have been corrected (Line 27 and 54).

Yours truly,

G.A. Akinlabi
